# DataLad: distributed system for joint management of code, data, and their relationship

**DOI:** 10.21105/joss.03262

**Published:** 2021-07-01

**Authors:** Yaroslav O. Halchenko, Kyle Meyer, Benjamin Poldrack, Debanjum Singh Solanky, Adina S. Wagner, Jason Gors, Dave MacFarlane, Dorian Pustina, Vanessa Sochat, Satrajit S. Ghosh, Christian Mönch, Christopher J. Markiewicz, Laura Waite, Ilya Shlyakhter, Alejandro de la Vega, Soichi Hayashi, Christian Olaf Häusler, Jean-Baptiste Poline, Tobias Kadelka, Kusti Skytén, Dorota Jarecka, David Kennedy, Ted Strauss, Matt Cieslak, Peter Vavra, Horea-Ioan Ioanas, Robin Schneider, Mika Pflüger, James V. Haxby, Simon B. Eickhoff, Michael Hanke

**Affiliations:** 1 Center for Open Neuroscience, Department of Psychological and Brain Sciences, Dartmouth College, Hanover, NH, USA; 2 Institute of Neuroscience and Medicine, Brain & Behaviour (INM-7), Research Center Jülich, Jülich, Germany; 3 McGill Center for Integrative Neuroscience, Montreal, Canada; 4 CHDI Management/CHDI Foundation, Princeton, NJ, USA; 5 Lawrence Livermore National Lab, Livermore, CA, USA; 6 Massachusetts Institute of Technology, Cambridge, MA, USA; 7 Stanford University, Stanford, CA, USA; 8 Quest Diagnostics, Marlborough, MA, USA; 9 The University of Austin at Austin, Austin, TX, USA; 10 Indiana University, Bloomington, IN, USA; 11 Institute of Systems Neuroscience, Medical Faculty, Heinrich Heine University Düsseldorf, Düsseldorf, Germany; 12 Faculty of Medicine and Health Sciences, McConnell Brain Imaging Center, McGill University, Montreal, Canada; 13 University of Oslo, Oslo, Norway; 14 University of Massachusetts Medical School, Worcester, MA, USA; 15 Montreal Neurological Institute, McGill University, Montreal, Canada; 16 University of Pennsylvania, Philadelphia, PA; 17 Department of Biological Psychology, Otto-von-Guericke-University Magdeburg, Magdeburg, Germany; 18 Department of Biological Engineering, Massachusetts Institute of Technology, Cambridge, USA; 19 Independent Developer, Germany; 20 Potsdam Institute for Climate Impact Research (PIK) e. V., Potsdam, Germany

## Abstract

DataLad is a Python-based tool for the joint management of code, data, and their relationship, built on top of a versatile system for data logistics (git-annex) and the most popular distributed version control system (Git). It adapts principles of open-source software development and distribution to address the technical challenges of data management, data sharing, and digital provenance collection across the life cycle of digital objects. DataLad aims to make data management as easy as managing code. It streamlines procedures to consume, publish, and update data, for data of any size or type, and to link them as precisely versioned, lightweight dependencies. DataLad helps to make science more reproducible and FAIR (Wilkinson et al., 2016). It can capture complete and actionable process provenance of data transformations to enable automatic re-computation. The DataLad project (datalad.org) delivers a completely open, pioneering platform for flexible decentralized research data management (RDM) (Hanke, Pestilli, et al., 2021). It features a Python and a command-line interface, an extensible architecture, and does not depend on any centralized services but facilitates interoperability with a plurality of existing tools and services. In order to maximize its utility and target audience, DataLad is available for all major operating systems, and can be integrated into established workflows and environments with minimal friction.

## Statement of Need

Code, data and computing environments are core components of scientific projects. While the collaborative development and use of research software and code is streamlined with established procedures and infrastructures, such as software distributions, distributed version control systems, and social coding portals like GitHub, other components of scientific projects are not as transparently managed or accessible. Data consumption is complicated by disconnected data portals that require a large variety of different data access and authentication methods. Compared with code in software development, data tend not to be as precisely identified because data versioning is rarely or only coarsely practiced. Scientific computation is not reproducible enough, because data provenance, the information of how a digital file came to be, is often incomplete and rarely automatically captured. Last but not least, in the absence of standardized data *packages*, there is no uniform way to declare actionable data dependencies and derivative relationships between inputs and outputs of a computation. DataLad aims to solve these issues by providing streamlined, transparent management of code, data, computing environments, and their relationship. It provides targeted interfaces and interoperability adapters to established scientific and commercial tools and services to set up unobstructed, unified access to all elements of scientific projects. This unique set of features enables workflows that are particularly suited for reproducible science, such as actionable process provenance capture for arbitrary command execution that affords automatic re-execution. To this end, it builds on and extends two established tools for version control and transport logistics, Git and git-annex.

### Why Git and git-annex?

Git is the most popular version control system for software development^1^. It is a distributed content management system, specifically tuned towards managing and collaborating on text files, and excels at making all committed content reliably and efficiently available to all clones of a repository. At the same time, Git is not designed to efficiently handle large (e.g., over a gigabyte) or binary files (see, e.g., Kenlon, 2016). This makes it hard or impossible to use Git directly for distributed data storage with tailored access to individual files. Git-annex takes advantage of Git’s ability to efficiently manage textual information to overcome this limitation. File content handled by git-annex is placed into a managed repository annex, which avoids committing the file content directly to Git. Instead, git-annex commits a compact reference, typically derived from the checksum of a file’s content, that enables identification and association of a file name with the content. Using these identifiers, git-annex tracks content availability across all repository clones and external resources such as URLs pointing to individual files on the web. Upon user request, git-annex automatically manages data transport to and from a local repository annex at a granularity of individual files. With this simple approach, git-annex enables separate and optimized implementations for identification and transport of arbitrarily large files, using an extensible set of protocols, while retaining the distributed nature and compatibility with versatile workflows for versioning and collaboration provided by Git.

### What does DataLad add to Git and git-annex?

#### Easy to use modularization.

Research workflows impose additional demands for an efficient research data management platform besides version control and data transport. Many research datasets contain millions of files, but a large number of files precludes managing such a dataset in a single Git repository, even if the total storage demand is not huge. Partitioning such datasets into smaller, linked components (e.g., one subdataset per sample in a dataset comprising thousands) allows for scalable management. Research datasets and projects can also be heterogeneous, comprising different data sources or evolving data across different processing stages, and with different pace. Beyond scalability, modularization into homogeneous components also enables efficient reuse of a selected subset of datasets and for recording a derivative relationship between datasets. Git’s *submodule* mechanism provides a way to nest individual repositories via unambiguously versioned linkage, but Git operations must still be performed within each individual repository. To achieve modularity without impeding usability, DataLad simplifies working with the resulting hierarchies of Git repositories via recursive operations across dataset boundaries. With this, DataLad provides a “monorepo”-like user experience in datasets with arbitrarily deep nesting, and makes it trivial to operate on individual files deep in the hierarchy or entire trees of datasets. A testament of this is datasets.datalad.org, created as the project’s initial goal to provide a data distribution with unified access to already available public data archives in neuroscience, such as crcns.org and openfmri.org. It is curated by the DataLad team and provides, at the time of publication, streamlined access to over 260 TBs of data across over 5,000 subdatasets from a wide range of projects and dozens of archives in a fully modularized way.

#### Re-executable annotation of changes.

Digital provenance is crucial for the trustworthiness and reproducibility of a research result, and contributes to the reusability aspect of the FAIR principles (Wilkinson et al., 2016). Knowing which code and data were used is essential, but, for changes that are programmatically introduced, how a command or script was invoked is another key piece of information to capture. One approach is to include this information in the Git commit message that accompanies a change, but doing so manually is tedious and error prone. To solve this, DataLad supports executing a command and automatically generating a commit message that includes a structured record with comprehensive details on the invocation. In addition to providing reliable information about past command-line invocations, these machine-readable records make it possible to easily re-execute commands (e.g., to verify if a result is computationally reproducible or to apply an analog change to a different dataset state).

#### Targeted interfaces and interoperability adapters.

Interoperability with scientific or commercial computing and storage services allows researchers to integrate data management routines into their established workflows with minimal friction. Git can already interact with other local or remote repositories via standard or custom network transport protocols. DataLad implements support for additional services that require custom protocols, such as the Open Science Framework (OSF) (Hanke, Poldrack, et al., 2021). Git-annex readily provides access to a wide range of external data storage resources via a large set of protocols. DataLad builds on this support and adds, for example, more fine-grained access (e.g. direct access to individual components contained in an archive hosted on cloud storage) or specialized services, such as XNAT (http://www.xnat.org/). Efficient and seamless access to scientific data is implemented using the *special remote* protocol provided by git-annex (Hess, 2013), through which external tools, like DataLad, can provide custom transport functionality transparently to a user. With this approach, DataLad and other projects can jointly facilitate access to an ever-growing collection of resources (Hess, 2011) and overcome technological limitations of storage solutions, like file size or inode limits.

#### Metadata management.

Metadata are essential for scientific discovery, as they are routinely used to complete all data analyses. Metadata is the core concept behind Git and git-annex functioning: Git records and uses metadata about each change (author, date, description, original state, etc) for each commit. Git-annex manages metadata about content availability and allows to associate additional arbitrary key-value pairs to any annexed content. Files managed by git and git-annex can in turn be of standardized file formats comprised of data with rich metadata records. Moreover, entire repositories might conform to a standard (e.g., BIDS (Gorgolewski et al., 2016)) or provide a standardized dataset descriptor (e.g., Frictionless data package). To facilitate metadata availability and utility, DataLad provides an extensible framework for metadata extraction and aggregation. Metadata for each file (contained in the file or recorded by git and git-annex) or associated with the entire dataset can be extracted into a collection of machine-readable (JSON) records and aggregated across all contained sub-datasets. Such simple mechanism makes it possible to provide immediate access to metadata about all contained data within a larger super-dataset (such as datasets.datalad.org).

## Overview of DataLad and its ecosystem

### Design principles

Besides the free software nature and open processes of the DataLad project, the development of DataLad is guided by four principles to ensure its open and domain agnostic nature, to maximize the long-term utility of its datasets and to minimize users’ technical debt:

- Datasets and the files they comprise are the only two recognized entities
- A dataset is a Git repository with an *optional* annex
- Minimization of custom procedures and data structures
- Complete decentralization, with no required central server or service, but maximum interoperability with existing 3rd-party resources and infrastructure

In conjunction, these principles aim to reduce the risk of adoption for DataLad users. They foster the resilience of an ecosystem using DataLad datasets as a standard package format for any digital objects by avoiding any critical dependency on service deployments governed by central entities, and even on DataLad itself, for access to any resources managed with DataLad.

### DataLad core

**Figure 1:**
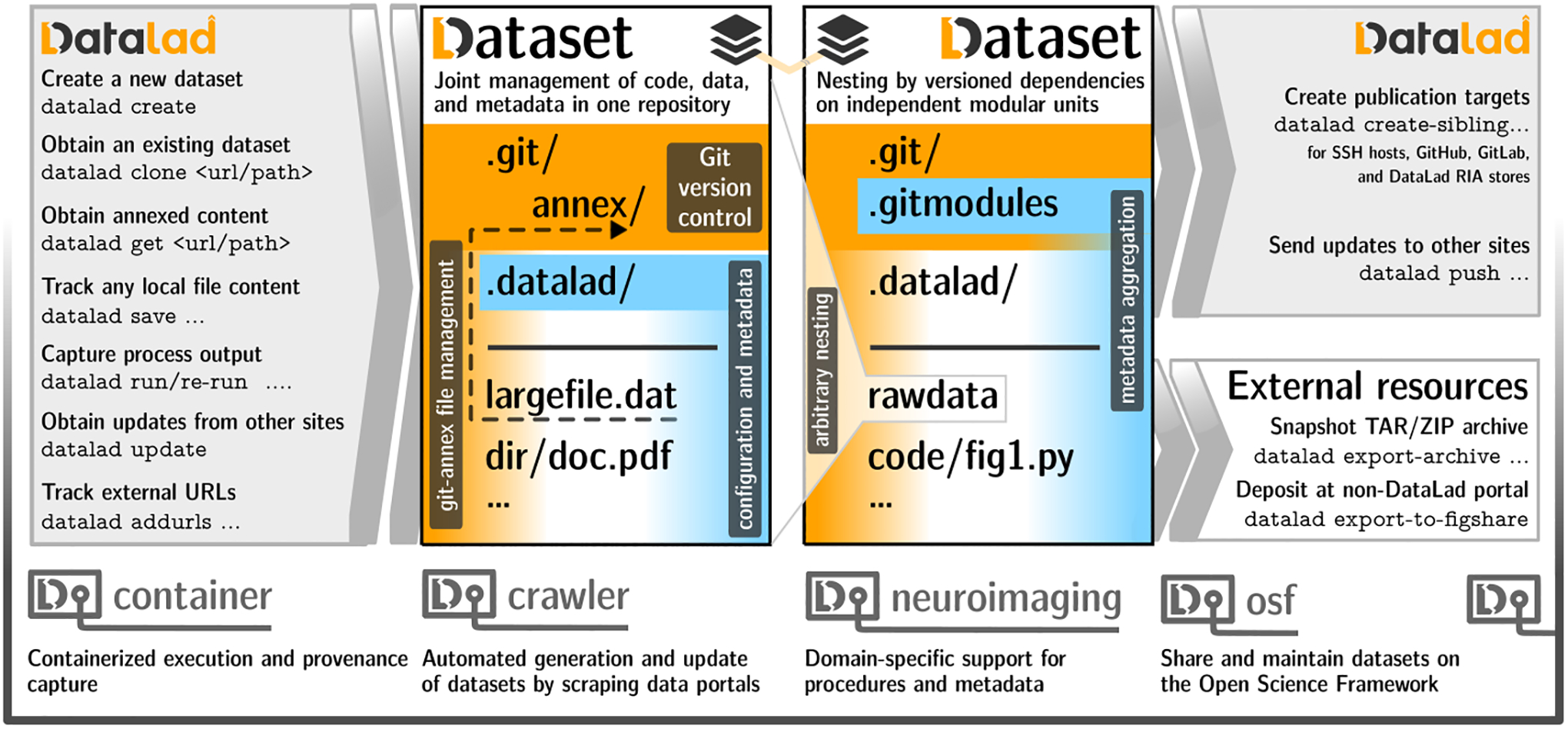
Schematic overview of a dataset, datasets nesting, and selected commands for content and dataset management. A more comprehensive cheatsheet is provided in the DataLad handbook (Wagner, 2020).

The datalad Python package provides both a Python library and a command line tool which expose core DataLad functionality to fulfill a wide range of decentralized RDM use cases for any domain. All DataLad commands operate on *DataLad datasets*. On a technical level, these datasets are Git repositories with additional metadata. On a conceptual level, they constitute an overlay structure that allows to version control files of any size, track and publish files in a distributed fashion, and record, publish, and execute actionable provenance of files and file transformations. Figure 1 summarizes key commands and concepts for local or distributed data and provenance management.

**Figure 2:**
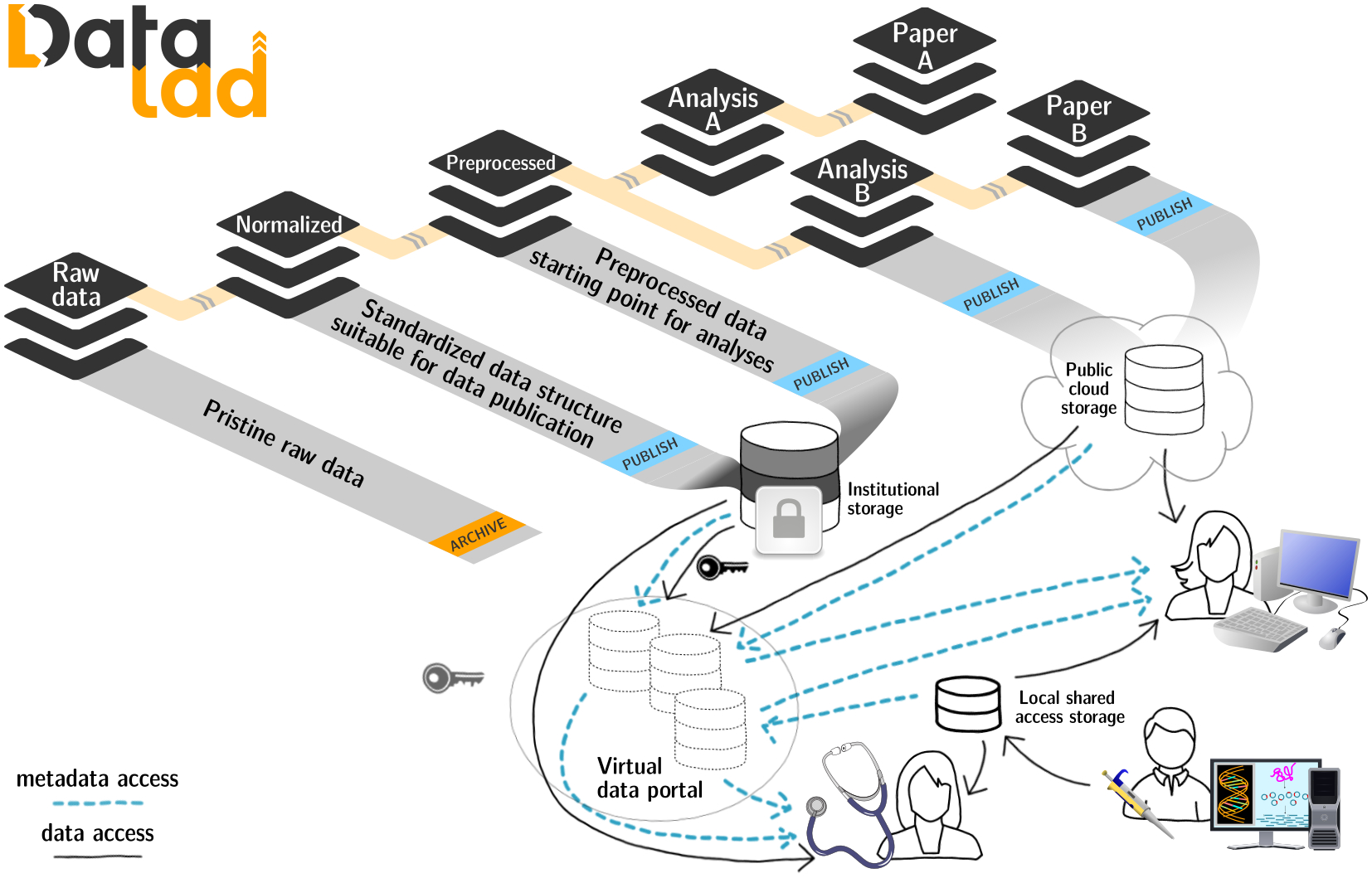
DataLad datasets are reusable modular components, which can be nested to establish a complete provenance trail all the way from a publication to the original data. Various access schemes to datasets and data are provided, and further extensibility is a key architectural property.

DataLad’s features can be flexibly integrated into standard scientific workflows. For example, by using the concept of dataset nesting to modularize the evolution of a research project, DataLad can fulfill the YODA principles for reproducible science (YODA Team, 2021), and, with this simple paradigm, facilitate efficient access, composition, scalability, reuse, sharing, and reproducibility of results (see Figure 2). With core commands that aim to simplify operation of the underlying tools, DataLad makes RDM workflows more accessible to novices and experts alike. Importantly, compatibility with all Git/git-annex functionality is retained.

### Extensions

Like Git and git-annex, DataLad core is a generic tool that is not specifically tuned to particular data types or use cases. It offers a robust foundation to build more specialized solutions on top of. *DataLad extensions*, stand-alone Python packages with additional DataLad functionality, extend DataLad with domain-focused or technology-specific features. A dedicated datalad-extension-template repository provides a starting point for creating new DataLad extensions. Some established extensions include:

- datalad-container (Meyer et al., 2021) to simplify management and use of Docker and Singularity containers typically containing complete computational environments
- datalad-crawler (Halchenko et al., 2021) to automate creation and updates of DataLad datasets from external resources
- datalad-neuroimaging (Hanke et al., 2020) to provide neuroimaging-specific procedures and metadata extractors
- datalad-osf (Hanke, Poldrack, et al., 2021) to collaborate using DataLad through the Open Science Framework (OSF)
- datalad-ukbiobank (Hanke, Waite, et al., 2021) obtain and BIDS-normalize imaging data releases of the UKBiobank

The same mechanism of extensions is used for rapid development of new functionality to later be moved into the core tool (e.g., datalad-metalad). The datalad-extensions repository provides a list of extensions and continuous integration testing of their released versions against released and development versions of the DataLad core.

### External uses and integrations

DataLad can be used as an independent tool to access and manage data (see e.g. Wittkuhn & Schuck (2021), Gautheron et al. (2021), Gautheron (2021)) or as a core technology behind another tool or a larger platform (e.g. Far et al. (2021)). TemplateFlow (Ciric et al., 2021) uses DataLad for the management of neuroimaging templates. OpenNeuro uses DataLad for data logistics with data deposition to a public S3 bucket. CONP-PCNO adopts aforementioned features for modular composition and nesting to deliver a rich collection of datasets with public or restricted access to data. ReproMan integrates with DataLad to provide version control and data logistics. www.datalad.org/integrations.html provides a more complete list of DataLad usage and integration with other projects, and Hanke, Pestilli, et al. (2021) provides a systematic depiction of DataLad as a system for decentralized RDM used by a number of projects.

### Documentation

Developer-focused technical documentation at docs.datalad.org, with detailed descriptions of the command line and Python interfaces, is automatically generated from the DataLad core repository. A comprehensive handbook (Wagner et al., 2021a) provides user-oriented documentation with an introduction to research data management, and numerous use case descriptions for novice and advanced users of all backgrounds (Wagner et al., 2021b).

### Installation

The handbook provides installation instructions for all major operating systems. DataLad releases are distributed through PyPI, Debian, NeuroDebian, brew, and conda-forge. The datalad-installer (also available from PyPI) streamlines the installation of DataLad and its dependencies, in particular git-annex, across a range of deployment scenarios, such as continuous integration systems, or high-performance computing (HPC) environments.

### Development

DataLad has been developed openly in a public repository (github.com/datalad/datalad) since its inception in 2013. At the time of this publication, the repository amassed over 13.5k commits, 2.5k merged PRs, and 2.3k closed (+700 open) issues from over 30 contributors. Issue tracker, labels, milestones, and pull requests are used to coordinate development. The development process of DataLad is not isolated from its foundational building blocks. For every new feature or bug fix the most appropriate software layer is determined to maximize the size of the benefitting user base and, importantly, also the associated developer audience. This strategy aims to achieve a robust integration with the larger open source software ecosystem, and simultaneously minimize the total technical debt carried solely by the DataLad development team. Consequently, DataLad development is tightly connected to and involves frequent communication with the git-annex project and its main developer Joey Hess (Hess & DataLad Team, 2016). To guarantee robust operation across various deployments, DataLad heavily utilizes continuous integration platforms (Appveyor, GitHub actions, and Travis CI) for testing DataLad core, building and testing git-annex (in a dedicated github.com/datalad/git-annex), and integration testing with DataLad extensions (datalad-extensions).

### Contributions

DataLad is free and open source software and encourages unconstrained use and reuse in any context. Therefore, DataLad is released under DFSG- and OSI-compliant MIT/Expat license. License terms for reused components in the code-base are provided in the COPYING file. The project aims to promote contributions rather than detached developments in forks and anyone is highly welcome to contribute to DataLad in any form under these terms. Technical and procedural guidelines for such contributions can be found in the CONTRIBUTING.md file shipped within DataLad’s source repository. Contributors are acknowledged on the project website, and also credited in the form of co-authorship in the Zenodo-based archival of software releases. All co-authors of this paper as well as the contributors acknowledged below have added to the project with code- or non-code-based contributions, and we thank past, present, and future contributors of this community for their involvement and work.

## Supplementary Material

10.21105.joss.03262.jats

## Footnotes

1
https://en.wikipedia.org/wiki/Git#Adoption
